# The role of the perception of partner support in sexual satisfaction and sexual quality of life of primigravidas

**DOI:** 10.1590/1806-9282.20240884

**Published:** 2025-03-31

**Authors:** Nermin Altunbaş, Birnur Yeşildağ

**Affiliations:** 1 Sivas Cumhuriyet University, Faculty of Health Sciences, Department of Nursing – Sivas, Turkey.; 2 Nigde Omer Halisdemir University, Zübeyde Hanım Faculty of Health Sciences, Department of Nursing – Niğde, Turkey.

**Keywords:** Life quality, Perceived social support, Pregnant women, Sexuality, Sexual satisfaction

## Abstract

**OBJECTIVE:**

The aim of this study was to determine the role of the perception of spousal support in sexual satisfaction and sexual quality of life of primigravidas.

**METHODS:**

This study used a descriptive and correlational design and consisted of 412 primigravidas. The target population of the study was pregnant women who presented to an obstetrics and gynecology clinic of a hospital located in a province on the Anatolian side of Turkey. Data were collected by face-to-face interviews using the "Personal Information Form," "Perception of Spousal Support in Pregnancy Scale," "New Sexual Satisfaction Scale," and "Sexual Quality of Life Scale-Female" between December 05, 2022 and March 31, 2023. The study included primigravida women, being above the age of 18 years, being in the last trimester of pregnancy, being married and living with their spouses, being literate, pregnant women and their spouses not having been previously diagnosed with sexual dysfunction, not having become pregnant with infertility treatment, and not diagnosed with a high-risk pregnancy..". The data obtained from the study were analyzed using the Statistical Package for Social Sciences version 27.0 program. Data analysis included numbers, percentage distributions, interquartile range, Pearson correlation analysis, and linear regression analysis. Statistical significance was accepted at p<0.05.

**RESULTS:**

A good level of positive correlation was found between the Perception of Spousal Support in Pregnancy Scale and New Sexual Satisfaction Scale (r=0.972; p<0.001) mean scores, between the Perception of Spousal Support in Pregnancy Scale and Sexual Quality of Life Scale-Female (r=0.967; p<0.001) mean scores, and between the New Sexual Satisfaction Scale and Sexual Quality of Life Scale-Female (r=0.947; p<0.001) mean scores of primigravida women. The perception of spousal support was found to explain 97% of sexual quality of life and sexual satisfaction during pregnancy (F=6628.666; R^2^=0.970).

**CONCLUSION:**

The increase in the perception of spousal support during pregnancy also increases primigravida women's sexual satisfaction and sexual quality of life.

## INTRODUCTION

Pregnancy is one of the important life periods affecting sexuality, an indispensable part of life^1^. Factors causing negative effects on sexual life during this period include physiological factors that cannot be changed; psychological and social value judgments such as ambivalent emotions and changes in body image; and sociocultural factors such as traditions and myths^2,3^. On the contrary, problems in sexual life during pregnancy are usually experienced by primigravida women and women in their first trimester. Sexual problems may decrease particularly in the last trimester as the woman adapts to pregnancy^4^.

Decreased sexual desire is the most common problem in sexual life due to biopsychosocial reasons during pregnancy. The literature has documented a decrease in sexual desire and frequency of sexual intercourse at ratios of 40% during pregnancy^5^. During pregnancy, women also experience trust problems such as fear of losing sexual attractiveness and partner's love and interest as well as experience infidelity^2,6^. These conditions were supported by some studies in the literature, in which women stated that sexual intercourse during pregnancy was initiated by their husbands, they had sexual intercourse to prevent their husbands’ infidelity, and they could not reach orgasm^1,7^. Decreased sexual desire due to various reasons is one of the causes of the negative effects on sexual satisfaction and sexual quality of life^5^. Relationships, communication, harmony, and support between couples are reported to have a potential effect in reducing the psychological and sociocultural negative effects of pregnancy on sexual life^8,9^.

The perception of adequate partner support is thought to be beneficial in many ways, such as improving sexual life, increasing positive communication between partners, having a healthier pregnancy, and increasing emotional and psychological satisfaction^1,10,11^. For this reason, pregnant women's perception of partner support level is considered to play an effective role in problems related to sexual satisfaction and sexual quality of life frequently seen during pregnancy. The evaluation of perceived partner support, sexual satisfaction, and sexual quality of life for a holistic evaluation of women during pregnancy could guide planning care and counseling. Besides, the lack of research examining the role of the perception of partner support on sexual satisfaction and sexual quality of life during pregnancy indicates an important gap in the literature and provides a guide for similar studies to be conducted in the future. Hence, this study aimed to determine the role of the perception of partner support in sexual satisfaction and sexual quality of life of primigravida women.

## METHODS

### Ethical considerations

Ethics committee approval was obtained from the Non-Interventional Clinical Research Ethics Committee of Sivas Cumhuriyet University, located in a province on the Anatolian side of Turkey, dated October 19, 2022, and numbered 2022-10/03. Institutional permission dated December 02, 2022, and numbered E-76728045-044 was obtained from the hospital where the forms would be applied. Publication ethics principles were followed at every stage of the study. All participants who agreed to participate in the study were informed about the study and were asked to sign an informed consent form.

### Study design

This study used a descriptive and correlational design.

### Target population and the sample

The target population of this study was pregnant women who presented to an obstetrics and gynecology outpatient clinic of a hospital located in a province on the Anatolian side of Turkey. The hospital is located in the city center and serves as a public institution affiliated with the State. There are 14 obstetrics and gynecology clinics in the hospital. The sample size was calculated using the formula for groups with a known population [n=N×t^2^×p×q/d^2^(N-1)+t^2^×p×q], where (N=862, t=1.96; p=0.50, q=0.50; and d=0.05), indicating a minimum of 267 participants. A total of 412 pregnant women who met the inclusion criteria and agreed to participate in the study were included in the sample group.

Some inclusion criteria were determined for including pregnant women in the sample group, such as being primigravida (the first pregnancy is important because it provides purer and unaffected data on women's perceptions of changes in their sexual lives and partner support, as they experience this experience for the first time), being aged above 18 years (required in terms of legal responsibility and maturity), being in the last trimester of pregnancy (physical and psychological changes related to pregnancy become more apparent during this period and the effects of these changes on sexual life are experienced most intensely), being married and not living with one's spouse (since the study examines the perception of partner support), being literate (important in terms of pregnant women being able to understand and evaluate the information provided during the research process), pregnant women and their spouses not having been previously diagnosed with sexual dysfunction (sexual satisfaction levels and sexual lives of individuals with sexual dysfunction may be affected by different factors), not having become pregnant with infertility treatment (these treatment processes may have different effects on sexual life both psychologically and physically), and not having been diagnosed with a high-risk pregnancy (high-risk pregnancies may negatively affect women's sexual life and perception of partner support).

### Data collection method

Data were collected by face-to-face interviews conducted between December 05, 2022, and March 31, 2023. A random sample of 858 pregnant women who presented to an outpatient clinic were evaluated using the question-answer method in line with the inclusion criteria. Pregnant women who met the criteria and agreed to participate voluntarily in the study were included in the sample. Before data collection, the primigravida women were given information about the purpose of the study and the forms, and their informed consent was obtained. They were asked to fill out the forms individually in the hospital environment. The data collection took about 15 min. The study was carried out with 412 pregnant women who filled out the forms completely, indicating that 48% of the population was reached.

### Data collection tools

Data were collected through the "Personal Information Form," the "Perception of Partner Support in Pregnancy Scale (PSSPS)," the "New Sexual Satisfaction Scale (NSSS)," and the "Sexual Quality of Life Scale-Female (SQOL-F)."

The Personal Information Form: This form included 10 questions about age, educational level, spouse's employment, economic condition, duration of the marriage, type of marriage, etc^2,8,10^.

The PSSPS: This scale developed by Yurdakul et al.^12^ consists of 16 items and three subscales, including cognitive support, emotional support, and financial support. Scores to be obtained from the scale range between 16 and 80, with higher scores indicating higher perceived partner support during pregnancy. The internal consistency Cronbach's alpha coefficient of the scale was reported to be 0.89. The Cronbach's alpha coefficient was found to be 0.98 in this study.

The NSSS: This scale, which was developed by Stulhofer et al.^13^, was adapted to Turkish by Tuğut^14^. The scale includes 20 questions rated on a 5-point Likert scale and has a self-centered subscale and spouse-partner/sexual activity-centered subscales. Scores to be obtained from the scale range between 20 and 100, with higher scores indicating good sexual satisfaction. The internal consistency Cronbach's alpha coefficient of the scale was reported to be 0.94. The Cronbach's alpha coefficient was found to be 0.96 in this study.

The SQOL-F: This scale, which was developed by Symonds et al.^15^, was adapted to Turkish by Tuğut and Gölbaşı^16^. The scale consists of 18 items rated on a 6-point Likert scale. Five items in the scale (items 1, 5, 9, 13, and 18) are coded reversely. Each item should be responded considering one's sexual life within the past 4 weeks. The total score obtained from the scale is converted to 100 using the formula: (raw score-18)×100/90. Higher scores obtained from the scale indicate a good sexual quality of life. The internal consistency Cronbach's alpha coefficient of the scale was reported to be 0.83. The Cronbach's alpha coefficient was found to be 0.75 in this study.

### Data analysis

Data analysis was performed using IBM SPSS Statistics for Windows, version 27.0 program. Descriptive features were presented as numbers, percentages, and interquartile range (IQR). Pearson correlation analysis and linear regression analysis were performed. Statistical significance was accepted as p<0.05.

## RESULTS

A total of 858 pregnant women were included in this study, of which 341 (39.7%) did not meet the inclusion criteria, 103 (12%) did not agree to participate in the study, and two (0.23%) did not fill out the forms completely and were not included in the sample.

Of all the participating 412 (48%) primigravida women, 36.4% were aged between 24 and 29 years (IQR=28 [24-33]), 59.5% were high school graduates, and 58.3% were employed. Of the spouses of the participating primigravida women, 37.9% were aged between 24 and 29 years (IQR=29 [25-33]) and 52.7% were high school graduates. It was found that 51% of the primigravida women had income equal to expenses. In addition, 90.3% had been married for 3 years or less (IQR=1.50 [1–2]), and 43.2% defined their marital relationship as good (Table 1).

**Table 1:** Introductory features of primigravida women (n=412).

Variables		n (%)
Age		
	18–23	95 (23.1)
	24–29	150 (36.4)
	30–35	125 (30.3)
	≥36	42 (10.2)
	Median (IQR)^a^	28 (24–33)
Educational level		
	Primary school	4 (1.0)
	Secondary school	14 (3.4)
	High school	245 (59.5)
	University	97 (23.5)
	Graduate	52 (12.6)
Occupational status		
	Yes	172 (41.7)
	No	240 (58.3)
Partner's age		
	18–23	65 (15.8)
	24–29	156 (37.9)
	30–35	134 (32.5)
	36–41	41 (10.0)
	≥42	16 (3.8)
	Median (IQR)^a^	29 (25–33)
Partners’ educational level		
	Primary school	4 (1.0)
	Secondary school	11 (2.6)
	High school	217 (52.7)
	University	112 (27.2)
	Graduate	68 (16.5)
Income status		
	Less than expenses	166 (40.3)
	Equal to expenses	210 (51.0)
	More than expenses	36 (8.7)
Duration of marriage		
	≤3 years	372 (90.3)
	≥4 years	40 (9.7)
	Median (IQR)^a^	1.50 (1–2)
Thoughts on the marital relationship		
	Intermediate	94 (22.8)
	Good	178 (43.2)
	Very good	140 (34.0)

a Interquartile range. IQR: interquartile range.

An analysis of the relationship between the scale mean scores indicated a good and positive correlation between PSSPS and NSSS (r=0.972; p<0.001), between PSSPS and SQOL-F (r=0.967; p<0.001), and between NSSS and SQOL-F (r=0.947; p<0.001) (Table 2).

**Table 2:** Relationship between Perception of Spousal Support in Pregnancy Scale, New Sexual Satisfaction Scale, and Sexual Quality of Life Scale-Female mean scores.

Scales		PSSPS	NSSS	SQOL-F
PSSPS	r	–	0.972	0.967
	p^a^	–	**<0.001**	**<0.001**
NSSS	r	0.972	–	0.947
	p^a^	**<0.001**	–	**<0.001**
SQOL-F	r	0.967	0.947	–
	p^a^	**<0.001**	**<0.001**	–

a Pearson correlation analysis.

Statistically significant values are denoted in bold. PSSPS: Perception of Spousal Support in Pregnancy Scale; NSSS: New Sexual Satisfaction Scale; SQOL-F: Sexual Quality of Life Scale-Female.

Linear regression analysis results showed that the perception of partner support in pregnant women was important in terms of sexual quality of life and sexual satisfaction. The perception of partner support significantly influenced the sexual quality of life (β=0.620; p=0.000) and sexual satisfaction (β=0.419; p=0.000). The perception of partner support was found to explain 97% of sexual quality of life and sexual satisfaction in pregnant women (F=6628.666; R^2^=0.970) (Table 3).

**Table 3:** The effect of the perception of partner support during pregnancy on the quality of sexual life and sexual satisfaction.

		B	SE	β	t	p	95%CI
Perception of partner support		-23.073	0.760		-30.368	**0.000**	-24.566–21.579
Quality of sexual life		0.655	0.015	0.620	44.412	**0.000**	0.626-0.684
Sexual satisfaction		0.646	0.022	0.419	29.971	**0.000**	0.603-0.688
R=0.985	F=28.666						
R^2^=0.970	p=0.000^a^						

a Linear regression analysis. Statistically significant values are denoted in bold. CI: confidence interval.

## DISCUSSION

This study investigated the role of spousal support perception on sexual satisfaction and the quality of sexual life in primigravida women. The study found that as the perception of spousal support increases, both sexual satisfaction and the quality of sexual life increase. It was also shown that the perception of spousal support has a significant effect on the sexual satisfaction and the quality of sexual life of primigravidas.

There are studies in the literature examining spousal support and relationship and evaluating sexual satisfaction and quality of sexual life. Bokaie et al.^17^ and García-Duarte et al.^18^ reported that primigravida women had moderate or lower levels of sexual satisfaction. In addition, Miranda et al.^9^ and Saniei et al.^5^ also suggested that there was a decrease in sexual satisfaction during pregnancy and that this was related to a lack of knowledge about sexuality. In some studies, it was reported that the quality of sexual life in primigravida women decreased or remained at a moderate level compared to pre-pregnancy^19–21^. These findings indicate that there is a decrease in sexual satisfaction and the quality of sexual life during pregnancy due to various reasons. It has also been reported that continuing sexual activity during pregnancy strengthens the relationship between couples and improves the quality of sexual life^22^. One study found that showing more empathic attention to the partner was linked to greater sexual satisfaction for first-time parents^23^. Rosen et al.^24^ emphasized that perceived partner sensitivity emerged as an important determinant of sexual satisfaction for new mothers. It was also stated that supporting new parents to respond to each other with care in their relationship would help increase sexual satisfaction during the transition to parenthood, which is already a vulnerable period. Ramsdell et al.^25^ stated that closeness and understanding between partners during pregnancy increases the quality of sexual life. Therefore, strengthening the relationship between partners and adequate perception of partner support can also increase sexual satisfaction and the quality of sexual life. Arisukwu et al.^7^ emphasized that elements such as emotional support, help with household chores, and financial support are especially important for pregnant women. Similarly, studies by Özbek and Beydağ^26^ and Yüksekal and Yurdakul^27^ also indicated that the perception of spousal support during pregnancy is high, but this perception decreases in high-risk pregnancies. In this study, a significant relationship was found between the perception of spousal support and sexual satisfaction and the quality of sexual life of the participating primigravida women. As the perception of spousal support increases, the quality of sexual life and satisfaction levels also increase. In addition, studies by Branecka-Woźniak et al.^3^ and Zare et al.^28^ emphasized the relationship between marital satisfaction and sexual satisfaction. Similarly, in our study, it was concluded that increasing the perception of spousal support can positively affect women's marital relationships and increase the quality of sexual life. Bourque-Morel et al.^29^ stated that fluctuations in sexual satisfaction during pregnancy can lead to attachment avoidance between spouses and therefore women need to be supported. Another study reported that the quality of marital relationships may affect the sexual satisfaction of primigravidas^5^. In addition, there are studies in the literature indicating that psychological factors such as depression, anxiety, and body image are also linked to spousal support. In Güner's^30^ study, it was observed that increasing the perception of spousal support reduced negative mental symptoms, such as depression and anxiety. In addition, Doğrul and Alan-Dikmen^31^ stated that the perception of motherhood increased as spousal support increased. These findings are consistent with the positive findings of our study on the relationship between spousal support and sexual satisfaction and reinforce the critical role of spousal support during pregnancy on general mental health and relationship quality. Yüksekal and Yurdakul^27^ and Tosun-Güleroğlu and Onat^32^ reported a relationship between pregnant women's perceptions of spousal support and age, educational level, family type, spouse's age, type of marriage, number of children, number of pregnancies, and desire for pregnancy. In conclusion, this study shows that the perception of spousal support plays an important role in sexual satisfaction and the quality of sexual life in primigravida women. Consistent with other studies in the literature, increasing spousal support may contribute positively to both the psychological well-being and sexual health of women during pregnancy. It is thought that open communication with couples about sexual health and relationship satisfaction, especially in the prenatal period, may help both pregnant women and their partners go through this process in a healthier and more supportive way^33,34^.

In this study, examining pregnant women only in the last trimester may not reflect the sexual satisfaction and perception of partner support experienced in different periods of pregnancy. Since the study only included married women living with their spouses, unmarried, separated, or divorced women were excluded from this study. This provides limited information on how the perception of partner support is shaped in different situations. Since the study included women who had not previously been diagnosed with sexual dysfunction, information on the perception of partner support and sexual satisfaction of women with sexual problems cannot be obtained. This makes the findings of the study limited in terms of generalizing to a wider population. Since the study did not include women who underwent infertility treatment, it was not possible to examine the effects of this process on sexual satisfaction and perception of partner support of women who had gone through this process.

### Limitations and strengths

This study has some limitations. Since the study only included primigravida women, the results cannot be generalized to multiparous women. The findings from the study cannot be generalized to other cultures. The perception of sexual life and partner support may differ according to cultural norms, beliefs, and social structure. The fact that the study relied largely on women's subjective statements may affect the accuracy and reliability of the data. Participants may not fully share their true feelings on personal and intimate issues such as sexual satisfaction due to social pressures or feelings of shame. Since the study collected data only at one time point, it does not show how the perception of partner support and sexual life satisfaction changed over time during pregnancy.

In addition, this study has some strengths. The study aims to fill an important gap in knowledge about women's sexual health during pregnancy by examining the effects of the perception of partner support on sexual satisfaction and quality of sexual life. This makes a valuable contribution to women's health and especially to increasing awareness about sexual health during pregnancy. Being primigravida can shape the changes in a woman's sexual life as well as her relationship with her partner; therefore, the specific population focused on adds originality to the study. Since women experience significant changes in their physical, emotional, and sexual health during the last trimester of pregnancy, it is very meaningful to examine the perception of partner support during this period. This is a period when pregnancy-related difficulties are felt most intensely, and sexual satisfaction and partner support become critical. Reaching 412 pregnant women makes the findings of the study more reliable and valid in terms of statistics. The large sample size increases the validity of the results on a larger group and allows the findings to appeal to more women.

## CONCLUSION

This study found that as the perception of spousal support increases in primigravida women, the sexual satisfaction and the quality of sexual life increase; the perception of spousal support has a strong and positive effect on both sexual satisfaction and quality of sexual life; and the perception of spousal support explains a large part of sexual satisfaction and quality of sexual life. These results emphasize that spousal support during pregnancy significantly affects the satisfaction levels and quality of life of women in their sexual lives. Therefore, spousal support can be considered an important component of sexual health during pregnancy.

In future studies, it is recommended that comparative, long-term follow-up studies be conducted to examine the effects of psychosocial interventions on couples during pregnancy, including other periods of pregnancy, and multiparous pregnant women from different cultures and living conditions.
